# Supplementary material for: Research on parameterized modeling and mechanical characteristics of shearer cables
Source: PLoS One. 2024 May 30;19(5):e0304007. doi: 10.1371/journal.pone.0304007 (PMC11139310; doi:10.1371/journal.pone.0304007)
Supplement: Attachment — Submitted filename: Review Comments.pdf [file pone.0304007.s001.pdf]

The study focuses on predicting the mechanical performance and fatigue life of shearer cables under harsh conditions. It addresses the challenges of parametric modeling and examines variations in pitch diameter ratios, stranding levels and monofilaments. Although this article provides useful information for modeling the structure of shearer cables, the following comments must be considered for the article to be accepted:

1. The parameters of the model shown in schematic Figures 2 and 3 are not sufficiently clear and have not been well defined and represented.
2. Considering existing models for shearer cable modeling, the innovation of the modeling presented in this article is questionable. Authors should transparently discuss the advantages and capabilities of Rhino Grasshopper modeling compared to previous works.
3. 3. Statistically, reliable determination of mechanical properties requires repeatability of tests under consistent conditions. Regarding Figure 11, explanations need to be added to the text regarding 4 repetitions of tensile tests for control KA6, 3 repetitions for control KA10 and 5 repetitions for Power 24*0.5 and Power 48*0.5. Presentation of the average experimental data along with the standard deviation in Tables 1 and 2 is also important. The standards, loading rates and load applications used in the tensile tests are not specified.
4. The characteristics of the bilinear isotropic hardening model are not provided in the numerical simulations!
5. The observed decreasing trend in stress values for Power Strand, Control Strand, and Ground Strand depicted in Figures 14 and 17 requires thorough interpretation and analysis to understand its significance in the context of the study.
6. The R-Squared value for the fitted curves in figure 18 should be reported.
7. Section 4-2 does not validate the fatigue life prediction results! How can we trust simulation results without validation? Further details on flexural fatigue loading should be provided. What was the mean stress and the stress amplitude? How was Ncode's predicted fatigue life corrected to 15.12e4 cycles and how was the 7% error determined?
8. In Figure 27, what were the tensile loading rates for 1 mm and 30 mm displacement? Why is the stress level in parallel stranding in the elastic stage at a low axial tension of 1 millimeter lower than in counter stranding? Why are the two types of twisting identical at a higher axial tension of 30 mm?
9. In summary, there is a lack of research reports on the optimized modeling parameters required to achieve the desired mechanical properties in shearer cables. This gap affects the completeness and applicability of the study's results in the field of shearer cable modeling and design.
